# Heritability of fear of humans in urban and rural populations of a bird species

**DOI:** 10.1038/srep31060

**Published:** 2016-08-08

**Authors:** Martina Carrete, Jesús Martínez-Padilla, Sol Rodríguez-Martínez, Natalia Rebolo-Ifrán, Antonio Palma, José L. Tella

**Affiliations:** 1 Department of Physical, Chemical and Natural Systems, Universidad Pablo de Olavide, Sevilla, Spain; 2 Department of Conservation Biology, Estación Biológica de Doñana, CSIC, Sevilla, Spain; 3 Department of Evolutionary Biology, Estación Biológica de Doñana, CSIC, Sevilla, Spain; 4 Centre d’Etudes Biologiques de Chizé (CEBC), UMR 7372 CNRS-Université de La Rochelle, Villiers-en-Bois, France; 5 Research Unit of Biodiversity – UMIB (CSIC/UO/PA), University of Oviedo, Spain; 6 Department of Biology, Biochemistry and Pharmacy, Universidad Nacional del Sur, Bahía Blanca, Argentina; 7 Departamento de Ecología, Genética y Evolución & IEGEBA-CONICET, Facultad de Ciencias Exactas y Naturales, Universidad de Buenos Aires, Buenos Aires, Argentina

## Abstract

Flight initiation distance (FID), a measure of an animal’s tolerance to human disturbance and a descriptor of its fear of humans, is increasingly employed for conservation purposes and to predict the response of species to urbanization. However, most work devoted to understanding variability in FID has been conducted at the population level and little is still known about inter-individual variability in this behaviour. We estimated the heritability of FID, a factor fundamental to understanding the strength and evolutionary consequences of selection of particular phenotypes associated with human disturbances. We used a population of burrowing owls (*Athene cunicularia*) monitored long-term and for which FID was previously shown to be highly consistent across an individual’s lifespan. Heritability estimates varied between 0.37 and 0.80, depending on the habitat considered (urban-rural) and method used (parent-offspring regressions or animal models). These values are unusually high compared with those previously reported for other behavioural traits. Although more research is needed to fully understand the underlying causes of this resemblance between relatives, selection pressures acting on this behaviour should be seriously considered as an important evolutionary force in animal populations increasingly exposed to human disturbance worldwide.

Human activities have transformed ecological patterns and processes around the world^1^, creating a wide range of unintentional experiments in which organisms either adapt to these changes or cease to exist when faced with severe and novel perturbations. Although traditionally the most adverse human activities for fauna have been related to natural landscape modification, a growing number of studies show that human presence *per se* can alter animal activities through behavioural changes^2^,^3^. Flight initiation distance (hereafter FID) is widely used as a quantitative measure of an animal’s tolerance to human disturbance^4^ and as a descriptor of its fear of humans^5^,^6^. FID has been largely employed as a species-specific trait useful for conservation purposes, such as in planning the recreational use of the countryside^7^,^8^,^9^ or predicting the response of species to urbanization^5^,^10^,^11^. A long-term monitoring study has recently shown that this behaviour is highly consistent across the individual’s lifespan of the burrowing owl *Athene cunicularia* (repeatability = 0.85–0.96), suggesting that the margin left for any existing inter-individual variance in plasticity would be very small^6^,^12^. Thus, the low fear of humans of some populations inhabiting anthropogenic habitats could not have resulted from individual habituation to human disturbance^9^,^13^,^14^,^15^,^16^ but to selective processes^5^,^10^,^17^. In this sense, it is compulsory to determine the additive genetic variation associated with the expression of this behavioural trait to understand the strength and evolutionary consequences of this selection. Although it has been shown that other risk-taking behaviours have a heritable component in vertebrates^18^ little is known about the fear of humans. The only study performed to date shows that the behavioural reactions of long-lived wandering albatross *Diomedea exulans* toward an approaching human are repeatable and heritable, with strong differences between breeding colonies^19^. However, most seabirds breed in remote places free of terrestrial predators, so they typically exhibit no instinctual fear of humans in their breeding colonies^9^. Moreover, the way that authors have measured fear is not the standard procedure used for FID, making extrapolations difficult.

Here, we estimated the heritability of FID in the burrowing owl in two adjacent habitats (urban and rural) differing in their degree of human disturbance. We used parent-offspring regressions and pedigree-based quantitative genetic models. Although there are numerous estimates of heritabilities for a wide range of traits, the great majority has been obtained for morphological and life-history traits and little is known about the heredability of behavioural traits^20^. To our knowledge, our results are the first to show that fear of humans, as measured by FID, is heritable in birds. Although more research is needed to fully understand the underlying causes of this resemblance between relatives, selection pressures acting on this behaviour should be seriously considered as an important evolutionary force for an increasing number of animal populations exposed to human disturbance.

## Results

**Figure 1:**
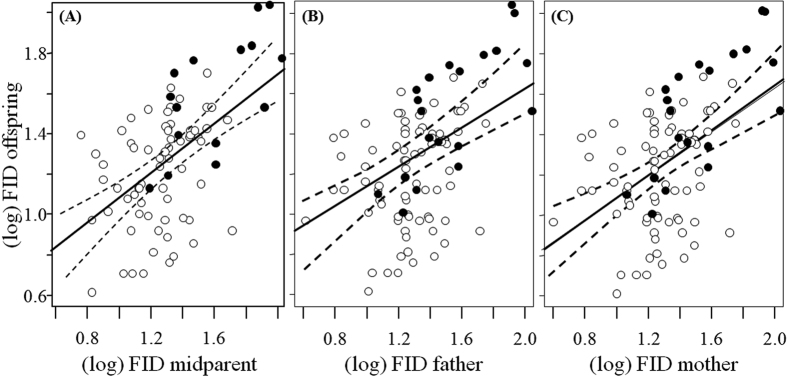
Offspring FID regressed (dashed lines: 95% confidence interval) on parent FID for burrowing owls. White and black dots represent urban and rural birds, respectively.

After measuring FID from 1748 individuals over eight years (2008–2015), we were able to establish 118 parent-offspring relationships (mother-offspring: FID range = 1–130 m, n = 96; father-offspring: FID range = 1–111 m, n = 103). Heritability (*h*^*2*^) of FID obtained through midparent-midoffspring regressions ranged from 0.15 to 0.8, without significant differences when calculated for the entire data set or separately for urban and rural birds (Table 1; Fig. 1A). Except for the urban father-offspring regression, all other mother- and father-offspring heritability estimates were significant and statistically similar, ranging from 0.37 to 0.80 (Table 1; Fig. 1B,C). Although heritabilities obtained from mother-offspring regressions were higher than those obtained from father-offspring regression, they were statistically equivalent (Table 1).

Animal models were performed using pedigree information from 1265 individuals, distributed through 3 generations. Heritability of FID using this method, which accounts for the significant lower FID of urban individuals (β = −0.42; 95% CI: −0.45–−0.39) and the larger FID of females (β = 0.06; 95% CI: 0.03–0.08), was 0.43 (95% CI: 0.19−0.60; heritabilities with models performed with different priors: 0.37−0.5; see Table S1 and Figure S1 for further details). The large 95% CI of this estimate suggests that it is not statistically different from heritability estimates obtained through parent-offspring regressions.

## Discussion

To understand the evolutionary consequences of human pressures on animal behaviours, it is fundamental to know whether behavioural responses have a genetic basis, i.e. if they are heritable between generations^21^. Here, we assessed whether FID, a behaviour widely used to measure anti-predator responses and fear of humans in birds^4^,^5^,^6^,^7^,^8^,^9^,^10^,^11^ and shown to be highly repeatable along an individual’s adulthood in our study species^6^, has a heritable component. Our estimates of heritability varied from 0.37 to 0.80, depending on the habitat (urban – rural) and method used (parent-offspring regressions or animal models), and are high compared with those previously reported for other heritable antipredator behaviours (0.12–0.42)^18^,^19^,^21^. While parent-offspring regressions have largely been used to estimate heritabilities in a variety of behaviours, animal models usually perform better because they are able to explain total phenotypic variance by partitioning residual variance into additive and non-additive genetic variance^22^,^23^,^24^. However, animal models require high quality data that are often difficult to obtain in wild populations. In our case, after marking more than 2,000 individuals for 10 consecutive years and measuring FID in more than 1,700 individuals for 8 of those years, we were able to use animal models to estimate heritability (*h*^*2*^ = 0.43). However, we obtained a rather large 95% CI, perhaps due to the low pedigree depth^25^. As this value is not different from those obtained using parent-offspring regressions, our results support the validity of regressions to estimate heritability when pedigree information is not available^26^.

Non-genetic inheritance provides a faster means of adapting to rapid environmental change than genetic inheritance alone^27^,^28^, with cultural evolution theoretically providing an important source of biodiversity through speciation^29^,^30^. In our case, the high fidelity of burrowing owls to their natal habitat (urban or rural, author’s unpublished data) does not allow us to properly assess to what extent non-genetic inheritance (i.e., epigenetic, ecological and cultural inheritance, and parental effects) could be contributing to our high heritability estimates^31^,^32^. Nonetheless, heritability estimates were high and statistically similar in both rural and urban habitats, which largely differ in levels of human disturbance, thus suggesting that these estimates were not largely inflated by the similar exposure to humans experienced by parents and their offspring. In any case, independent of the mechanisms promoting the significant resemblance between relatives, our results indicate that selection acting on this behaviour can have long-term consequences for animal populations^36^. More research is thus needed using other study models to properly generalize and fully understand the consequences of population and individual variability in this behaviour.

Selection of particular phenotypes (in this case, behaviours) associated with human disturbances^5^,^10^ can have profound effects on populations. Several studies have shown that risk-taking behaviours, within which we can include fear of humans, are actually correlated with exploration and aggression^33^,^34^,^35^,^36^, and linked to life history traits^37^. Thus, selection of human-tolerant phenotypes can have important ecological and evolutionary implications due to the inherent trade-offs of a syndrome structure^38^. Human presence in the wild selecting positively for individuals with a low fear of humans would result in a population with more exploratory and aggressive individuals, thus changing intraspecific interactions among them^35^,^36^. Moreover, demographic parameters of those individuals can be different^39^, and potential consequences such as the non-random dispersal of individuals between humanized and non-humanized habitats, should be taken into account as an unappreciated ecological and evolutionary force^5^,^17^,^40^. A similar scenario of human-induced evolution has been shown for fisheries, as harvesting has imposed a directional and disruptive selection process to key life-history traits genetically or phenotypically correlated with a suite of interrelated physiological, behavioral, and morphological characters^41^,^42^. In this sense, even when human activities such as habitat transformation, persecution, pollution or species introductions have been claimed as the main drivers of species loss^43^, human presence *per se* should be considered as another, maybe less obvious but potentially important, cause of biodiversity change.

## Methods

### Study species and study area

The burrowing owl is found across American open landscapes, showing diurnal activity and nesting in burrows excavated by the owls or by mammals^44^. Breeding pairs are easily located within the surrounding of their nests (usually 30 m) due to their territorial and highly conspicuous behaviour during the daytime^6^,^12^,^17^.

Our study site encompasses ca. 5,500 km^2^, including rural and urban areas around Bahía Blanca (Argentina). Rural habitats are mainly represented by large expanses of natural grasslands and pastures used for wide ranging livestock and low-intensive cereal crops. Human presence is extremely low and mostly restricted to a few paved or unpaved roads (with 0–0.1 pedestrians/h and 0.34–2.4 cars/h), so most owls breeding in rural habitats have little or even no close contact with humans (or only with the researchers). This strongly contrasts with urban owls, which excavate their nests in small (usually 0.01–0.1 ha) private and public gardens in urbanized residential areas, unbuilt spaces among houses, curbs of streets and even along large avenues. These owls are in constant contact with garden and house owners, children, pedestrians and intense car traffic. There is no clear habitat interface between urban and rural habitats, since urbanized areas are immediately surrounded by rural ones, and all birds sampled for FID unambiguously bred in urban or rural areas (for more details on the study area, human pressure and owl population, see refs 5,6,12,17,45).

### Field procedures

We trapped 950 adults and 1,245 chicks from 2006 to 2015, using bow nets and ribbon carpets, to mark them with a plastic numbered colour-ring readable at distance. Pair members were first sexed based on plumage patterns and colouration, and then confirmed by molecular analyses^18^. FIDs were measured in a sample of 791 banded adult owls during the late breeding season stage, i.e. when they were rearing chicks (from late November to late January), in 2008–2015. We enlarged sample sizes by measuring FID in another 957 non-ringed birds. These birds were breeding adults for which we were able to ring their offspring and/or their mates, so we could confirm their parent-offspring and mate relatedness despite being unmarked. Due to the high within-individual repeatability of FID recorded both within breeding seasons (r = 0.84–0.92)^12^ and across breeding seasons covering the lifespan of individuals (r = 0.90–0.96)^6^, we used one FID per individual or the mean when more than one value was available (see ref. 15 for the same approach). The standard procedure used was to walk towards undisturbed focal individuals, which were perched on the ground or on small poles close to their nests, following a direct trajectory at a constant speed of 0.5 m/s, with no obstacles between the bird and the observer. Distances at which birds fled were measured using a laser telemeter incorporated into 10 × 42 binoculars (Leica Geovid, range: 10–1300 m) or counting paces for distances of less than 10 m^5^,^6^,^12^. FIDs were measured during the day, when owls were easily located at distance, given the bare ground and short vegetation surrounding their nests. As shown in previous studies of this burrowing owl population^5^,^6^,^17^, urban individuals included here for heritability analyses also showed shorted FIDs (median 17 m, range 1–55 m, n = 252) than their rural counterparts (median = 32 m, range = 10–130 m, n = 65) (GLM on log-transformed FID, F = 70.24, p < 0.0001).

### Statistical analysis

Heritability (*h*^*2*^) of (log) FID was obtained from the slope of the regression of midoffspring on midparent and twice the slopes of midoffspring on each single-parent through GLMs with normal error distribution and identity link function, using the average value of full sibs to avoid pseudo-replication (see Figure S2 for actual values of parent-offspring FIDs). As heritability could differ between habitats, we obtained *h*^*2*^ for urban and rural birds separately as well as for all birds together. The different heritabilities obtained were compared by calculating z scores as (x_i_ − x_j_)/√(SE_i_^2^ + SE_j_^2^), where x_i_ and x_j_ are the two different estimates, and SE_i_ and SE_j_ the respective standard errors. Assortative mating does not affect midparent–offspring regressions, but it increases the regression of offspring on single parents by a factor (1 + *r*), where *r* is the phenotypic correlation between mates^46^. Breeders did not mate at random regarding FID^12^ so heritability values obtained from father/mother-offspring regressions were corrected for (urban birds: r = 0.61, n = 293 breeding pairs; rural birds: r = 0.72, n = 264 breeding pairs; all birds: r = 0.78, n = 557 breeding pairs; all p < 0.0001).

We ran animal models to estimate the heritability of FID (log-transformed) by using a Bayesian Markov chain Monte Carlo (MCMC) technique implemented in the MCMCglmm package in R^47^. Animal models are a type of mixed effect model, which estimates quantitative genetic parameters of a trait by assessing the phenotypic covariance between all pairs of relatives in the pedigree^48^. MCMC pedigree-based models are particularly useful to partition phenotypic variance into additive and non-additive genetic variance of phenotypes with Gaussian and non-Gaussian distributions, while simultaneously accounting for any potentially confounding effects. We assessed heritability as *V*_*A*_*/V*_*P*_, where *V*_*A*_ is the additive genetic variance and *V*_*P*_ the total phenotypic variance of FID, calculated as *V*_*A*_ + *V*_*R*_. In the ‘animal model’, *V*_*A*_ and *V*_*P*_ were inferred from the pedigree information, including both father and mother identities, and considering the effect of habitat and individual sex as fixed effects. Since FID is highly repeatable across the life span of burrowing owls^6^, we did not include year as a controlling variable in models. We used an uninformative prior for the fixed and random variances (V = 1, nu = 0.02^24^; but see Appendix 1 for results with other priors) for 1,000,000 iterations, preceded by a burn-in of 10,000 iterations and storing estimates of every 200th iteration to reduce autocorrelation. This provides us with the posterior probability density functions for the fixed term and all variance components, and thereby with the most probable size of each variance component, as well as its 95% credible interval (CI). We tested the statistical support of the fixed effect (habitat) by evaluating the extent to which their posterior distributions (95% CI) overlapped zero.

Parent-offspring regressions as well as animal models may underestimate heritability if social parents are not the genetic parents of all or some of the offspring^49^. However, burrowing owls are territorial birds that can be mostly considered as genetically monogamic, since extra-pair fertilizations and brood parasitism are infrequent even when individuals breed at very high densities in urban sites^45^.

### Ethics statements

Capture, banding and FID measures of burrowing owls were conducted under permits from the Argentinean wildlife agency (22500-4102/09), and the owners of private properties, in accordance with the approved guidelines of the Ethics Committee of CSIC (CEBA-EBD-11-28).

## Additional Information

**How to cite this article**: Carrete, M. *et al.* Heritability of fear of humans in urban and rural populations of a bird species. *Sci. Rep.* **6**, 31060; doi: 10.1038/srep31060 (2016).

## Supplementary Material

Supplementary Information

## Figures and Tables

**Table 1:** Heritability (*h* ^*2*^) of FID and its standard error (SE) estimated from the midparent-midoffspring and mother/father-offspring regressions for urban and rural burrowing owls.

Parent-offspring	Rural	Urban	Total
h^2^	0.69^***^	0.39^**^	0.64^****^
SE	0.22	0.14	0.11
n	15	66	81
	**rural-urban**	**rural-total**	**urban-total**
z	1.15	0.20	−1.40
P	0.2501	0.8415	0.1615
**father-offspring**†	**rural**	**urban**	**total**
h^2^	0.75^**^	0.15^ns^	0.43^***^
SE	0.37	0.22	0.19
n	20	83	103
	**rural-urban**	**rural-total**	**urban-total**
z	1.40	0.78	−0.97
P	0.1615	0.4354	0.3320
**mother-offspring**†	**rural**	**urban**	**total**
h^2^	0.80^**^	0.37^*^	0.63^****^
SE	0.36	0.26	0.22
n	20	76	96
	**rural-urban**	**rural-total**	**urban-total**
z	0.97	0.40	−0.76
P	0.3320	0.6892	0.4473
	**father-mother rural**	**father-mother urban**	**father-mother**
z	−0.10	−0.65	−0.69
P	0.9203	0.5157	0.4902

Significance of the estimate is shown (****P < 0.0001; ***P < 0.001; **P < 0.01; *P < 0.05; ns: P > 0.05). Heritability values are compared through z-test (z). n: sample size.

^†^Values corrected for assortative mating: r_urban birds_ = 0.61, r_rural birds_ = 0.72, r_all birds_ = 0.78.
