# Author Correction: Heritability of fear of humans in urban and rural populations of a bird species

**DOI:** 10.1038/s41598-023-32914-9

**Published:** 2023-04-12

**Authors:** Martina Carrete, Jesús Martínez-Padilla, Sol Rodríguez-Martínez, Natalia Rebolo-Ifrán, Antonio Palma, José L. Tella

**Affiliations:** 1 Department of Physical, Chemical and Natural Systems, Universidad Pablo de Olavide, Sevilla, Spain; 2 Department of Conservation Biology, Estación Biológica de Doñana, CSIC, Sevilla, Spain; 3 Department of Evolutionary Biology, Estación Biológica de Doñana, CSIC, Sevilla, Spain; 4 Centre d’Etudes Biologiques de Chizé (CEBC), UMR 7372 CNRS-Université de La Rochelle, Villiers-en-Bois, France; 5 Research Unit of Biodiversity – UMIB (CSIC/UO/PA), University of Oviedo, Oviedo, Spain; 6 Department of Biology, Biochemistry and Pharmacy, Universidad Nacional del Sur, Bahía Blanca, Argentina; 7 Departamento de Ecología, Genética Y Evolución & IEGEBA-CONICET, Facultad de Ciencias Exactas Y Naturales, Universidad de Buenos Aires, Buenos Aires, Argentina

Correction to: *Scientific Reports* 10.1038/srep31060, published online 8 August 2016

The original version of this Article contains errors in the Acknowledgements section.

“We thank N. Lois, S. Briones, M. Santillán, P. Laiolo, M. de la Riva, N. Tella-Carrete and M. Vázquez for their help in capturing and monitoring owls over the years. Field work was conducted under permits from Argentinean wildlife agencies and the owners of private properties. This work was supported by Canal Sur TV, Fundación Repsol, Projects CGL2012-31888 and CGL2015-71378 from MEC and COOPA20049 from CSIC (Spain). N.R.I. and S.R.M. were supported by CONICET (Argentina), A.P. by a La Caixa - Severo Ochoa foundation, J.M.P. by CGL2015-70639-P (Ministerio Economía y Competitividad, Spain) and M.C. by a Ramón y Cajal contract (RYC-2009-04860) and a contract of the UPO. S. Young revised the English.”

should read:

“This work was supported by CGL2015-71378 MINECO/FEDER, UE, Canal Sur TV, Fundación Repsol, Project CGL2012-31888 CGL2015-71378 MITECO, and COOPA20049 from CSIC (Spain). N.R.I. and S.R.M. were supported by CONICET (Argentina), A.P. by a La Caixa - Severo Ochoa foundation, J.M.P. by CGL2015-70639-P (Ministerio Economía y Competitividad, Spain) and M.C. by a Ramón y Cajal contract (RYC2009-04860) and a contract of the UPO. S. Young revised the English. We thank N. Lois, S. Briones, M. Santillán, P. Laiolo, M. de la Riva, N. Tella-Carrete, and M. Vázquez for their help in capturing and monitoring owls over the years. Field work was conducted under permits from Argentinean wildlife agencies and the owners of private properties.”

