# Supplementary material for: Heritability of fear of humans in urban and rural populations of a bird species
Source: Sci Rep. 2016 Aug 8;6:31060. doi: 10.1038/srep31060 (PMC4976307; doi:10.1038/srep31060)

Heritability of fear of humans in urban and rural populations of a bird species

Martina Carrete[1,2,*], Jesús Martínez-Padilla,[2,3], Sol Rodríguez-Martínez[4], Natalia Rebolo-Ifrán[5], Antonio Palma[2], and José L. Tella[2]

[1]Department of Physical, Chemical and Natural Systems, Universidad Pablo de Olavide, Sevilla, Spain

[2]Department of Conservation Biology, Estación Biológica de Doñana, CSIC, Sevilla, Spain.

[3]Research Unit of Biodiversity – UMIB (CSIC/ UO/PA), University of Oviedo, Spain.

[4]Department of Biology, Biochemistry and Pharmacy, Universidad Nacional del Sur, Bahía Blanca, Argentina

[5]Departamento de Ecología, Genética y Evolución & IEGEBA-CONICET, Facultad de Ciencias Exactas y Naturales, Universidad de Buenos Aires, Buenos Aires, Argentina

*Corresponding author: martina@ebd.csic.es

Appendix 1

We ran MCMCglmms with different priors (ie, different V and nu values) to test the sensitivity of our heritability estimates to them. Each group of models (ie, models sharing the same prior) were built considering the potential effects of sex and habitat on (log)FID. Models were ranked based on DIC, smaller values suggesting a better model. As commonly performed with AIC (Akaike Information Criterion), ΔDIC < 2 suggests alternative models.

Table S1. MCMCglmm built to calculate heritability of fear of humans in the burrowing owl *Athene cunicularia*. Heritability (h^2) and estimates of fixed effects (β) are shown with their 95% credible intervals (95% CI) and effective sizes (ES). DIC: Deviance Information Criterion. ΔDIC: $DIC_{model} - DIC_{best\ model}$.

Model	Prior	DIC	ΔDIC	h^2	95% CI	ES	Fixed effects	β	95% CI	ES
(log)fid~habitat+sex	V=1, nu=0.002	205.58	0.00	0.37	0.18 - 0.62	1974	habitat(urban)	-0.42	-0.45 - -0.39	4950
							sex(female)	0.06	0.03 - 0.08	4834
(log)fid~habitat		221.83	16.26	0.39	0.17 - 0.61	2010	habitat(urban)	-0.42	-0.45 - -0.4	4950

(log)fid~sex		240.18	34.60	0.78	0.60 - 0.93	1650	sex(female)	0.06	0.03 - 0.09	4735
(log)fid~none		231.86	26.29	0.77	0.61 - 0.94	1792				
(log)fid~habitat+sex	V=1, nu=0.02	202.44	0.00	0.43	0.19 - 0.60	2502	habitat(urban)	-0.42	-0.45 - -0.39	4950
							sex(female)	0.06	0.03 - 0.08	4950
(log)fid~habitat		213.97	11.53	0.41	0.21 - 0.62	2565	habitat(urban)	-0.42	-0.45 - -0.39	4950
(log)fid~sex		229.06	26.62	0.74	0.61 - 0.92	2308	sex(female)	0.06	0.02 - 0.09	4950
(log)fid~none		222.90	20.46	0.79	0.60 - 0.91	2375				
(log)fid~habitat+sex	V=1, nu=1	164.89	0.00	0.47	0.37 - 0.59	4950	habitat(urban)	-0.42	-0.45 - -0.39	4950
							sex(female)	0.06	0.03 - 0.09	4950
(log)fid~habitat		178.86	13.97	0.49	0.37 - 0.59	4950	habitat(urban)	-0.42	-0.06	4950
(log)fid~sex		277.62	112.73	0.62	0.51 - 0.74	4703	sex(female)	0.06	0.03 - 0.09	4950
(log)fid~none		287.23	122.34	0.6	0.50 - 0.73	4547				
(log)fid~habitat+sex	V=1, nu=1.002	164.98	0.00	0.46	0.37 - 0.59	4950	habitat(urban)	-0.42	-0.45 - -0.39	4600
							sex(female)	0.06	0.03 - 0.08	4950
(log)fid~habitat		178.97	13.99	0.48	0.36 - 0.59	4786	habitat(urban)	-0.42	-0.45 - -0.39	4950
(log)fid~sex		276.41	111.43	0.64	0.49 - 0.73	4555	sex(female)	0.06	0.03 - 0.09	5189

(log)fid~none		285.06	120.08	0.62	0.51 - 0.74	4513					
(log)fid~habitat+sex	V=1, nu=1000	3817.42	0.40	0.5	0.48 - 0.52	4950	habitat(urban)	-0.42	-0.52 - -0.31	4477
							sex(female)	0.06	-0.04 - 0.16	5165
(log)fid~habitat		3817.02	0.00	0.5	0.48 - 0.52		habitat(urban)	-0.42	-0.52 - -0.32	4677
(log)fid~sex		3873.61	56.60	0.5	0.48 - 0.52	4950	sex(female)	0.06	-0.04 - 0.16	4950
(log)fid~none		3873.23	56.21	0.5	0.48 - 0.52	4437				
(log)fid~habitat+sex	V=matrix(p.var/2), n=1	195.16	0.00	0.42	0.23 - 0.60	2760	habitat(urban)	-0.42	-0.45 - -0.39	5269
							sex(female)	0.06	0.03 - 0.08	4689
(log)fid~habitat		210.10	14.94	0.41	0.22 - 0.61	2927	habitat(urban)	-0.42	-0.45 - -0.39	4950
(log)fid~sex		229.56	34.40	0.78	0.60 - 0.90		sex(female)	0.06	0.03 - 0.09	4950
(log)fid~none		214.52	19.35	0.78	0.58- 0.89					

Figure S1. Heritabilities (h^2) of fear of humans in burrowing owls and their credible intervals obtained using different priors.

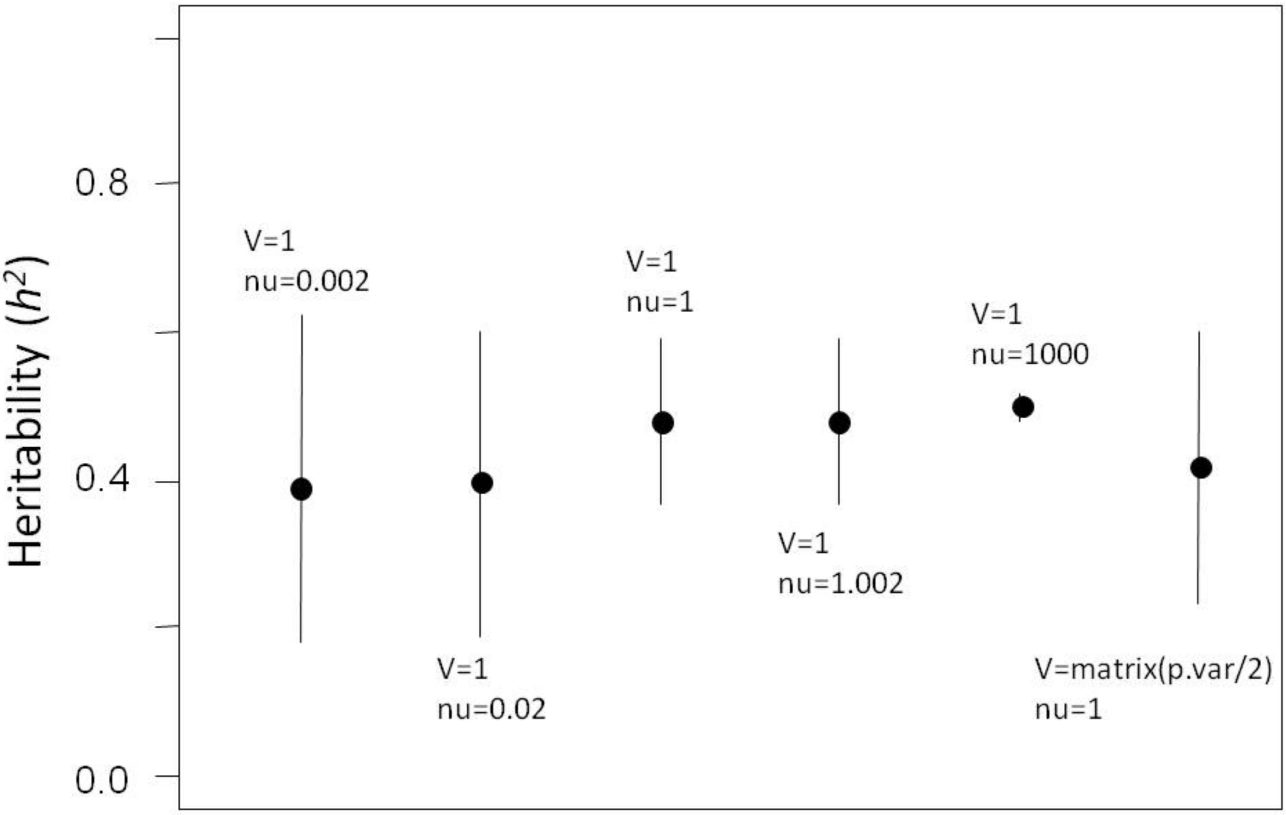

Considering that priors have no significant effects on heritability estimates, we calculate the additive genetic and the phenotypic variance (V_A and V_P, respectively) of fear of humans. Models were run for all birds together and for urban and rural birds separately (Table S2).

Table S2. Models ran to estimate the additive genetic and the phenotypic variance (V_A and V_P, respectively) of fear of humans for all birds together and separately for urban and rural birds, using an uninformative prior for the fixed and random variances (V=1, nu=0.02).

Model	V_A	95%CI	V_P	95%CI
All bird	0.03	0.02 – 0.05	0.08	0.07 – 0.08
Urban birds	0.04	0.01 - 0.05	0.07	0.07 - 0.08
Rural birds	0.05	0.02 - 0.08	0.08	0.07 - 0.09

Figure S2. FID measured on offspring and parent (father and mother) burrowing owls. White and black dots represent urban and rural birds, respectively.

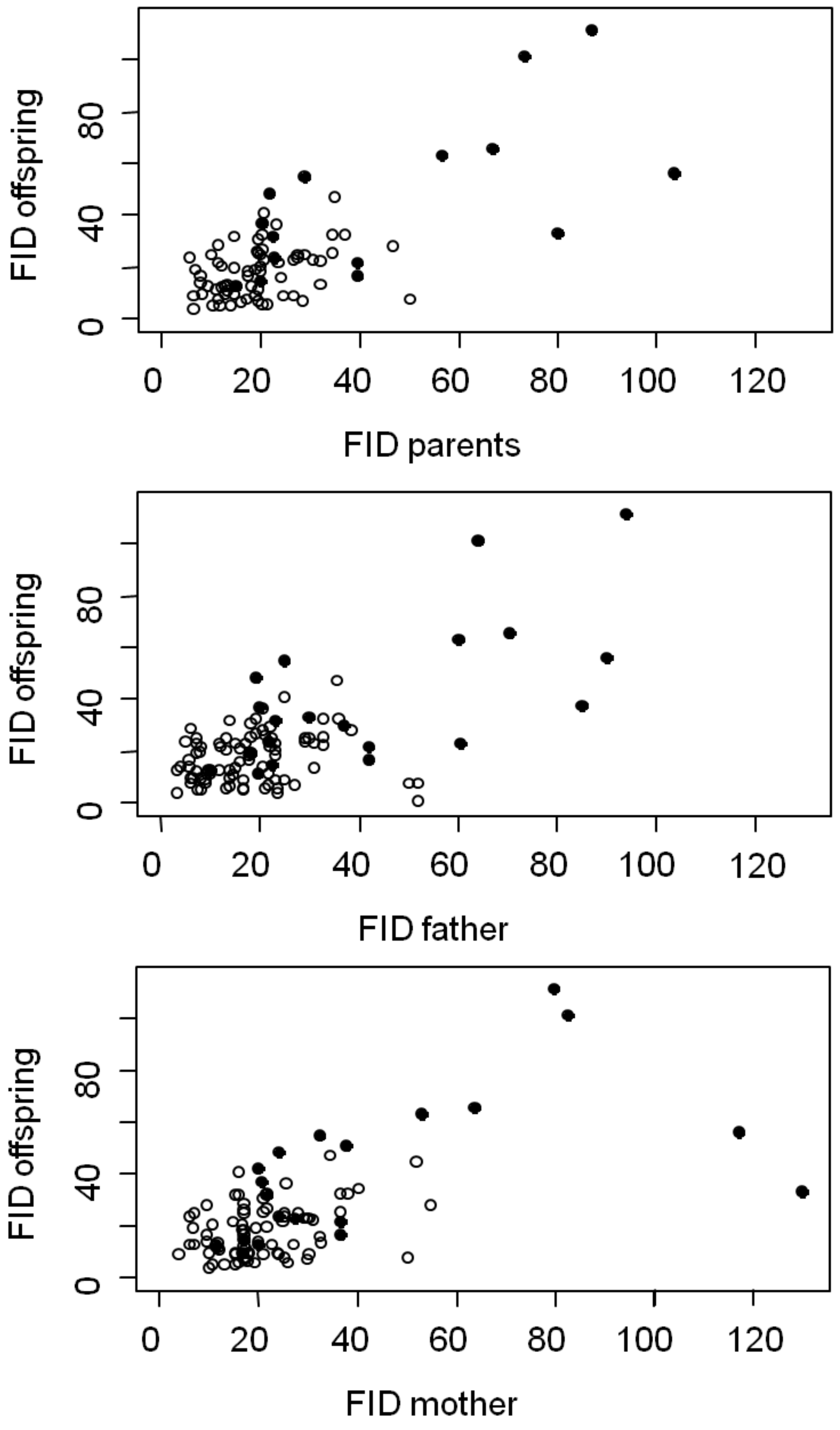

Supplement: Supplementary Information [file srep31060-s1.pdf]
